# BUILDING CLINICAL JUDGMENT IN THE HEALTH CARE WORKFORCE BY ADDRESSING THE 4MS AND MULTICOMPLEXITY

**DOI:** 10.1093/geroni/igad104.0136

**Published:** 2023-12-21

**Authors:** Margaret Avallone, Elyse Perweiler, Jennifer DeGennaro, Marilyn Mock

**Affiliations:** Rutgers University-Camden School of Nursing, Camden, New Jersey, United States; Virtual Health College of Medicine & Life Sciences of Rowan University - Rowan-Virtua School of Osteopathic Medicine, Stratford, New Jersey, United States; Rowan-Virtua SOM / NJISA, Stratford, New Jersey, United States; Fair Share Support Services, Inc., Camden, New Jersey, United States

## Abstract

Since 2019, NJGWEP, Rutgers School of Nursing-Camden and Fair Share Support Services, Inc. partnered in an affordable housing site in an underserved, disadvantaged community to support aging in place. Senior nursing students conduct Resident Health Risk Assessments (RHRAs) using the 4Ms framework to build assessment skills, sharpen clinical judgment, and address the Geriatric 5th M in a multi-complex resident population. Nursing students, working in teams with social service and community health workers, completed 92 RHRAs incorporating the 4Ms (What Matters, Mind, Medication, Mobility). On average, residents had > 3 risk factors related to the 4Ms (M=3.31); 25% +screen for depression, 25% +screen for cognitive impairment, 64% w/mobility deficits, 83% w/polypharmacy, and 80% on 1 or more high-risk medications. There were 53% w/diabetes, 65% w/hypertension and other biopsychosocial factors related to social determinants of health; 91% could be characterized as multi-complex (the Geriatric 5th M) and in need of services/supports to age in place. The 4Ms framework, with the addition of the 5th M, provides curricular structure for development of clinical judgment through focused assessment, analysis, flagging of key assessment data, prioritization of problems, and developing person-centered plans of care. Interprofessional (IP) case studies of increasing levels of difficulty experienced by affordable housing residents build cultural competence, foster development of critical thinking, clinical reasoning, and, when supplemented by weekly IP case reviews, facilitate shared decision-making and care planning. The process is further supported by a customized electronic database that permits monitoring person-centered recommendations and outcomes over time, gauging impact.

